# MMP2 and MMP7 at the invasive front of gastric cancer are not associated with mTOR expression

**DOI:** 10.1186/s13000-015-0449-z

**Published:** 2015-12-12

**Authors:** Jan Bornschein, Tina Seidel, Cosima Langner, Alexander Link, Thomas Wex, Michael Selgrad, Doerthe Jechorek, Frank Meyer, Elizabeth Bird-Lieberman, Michael Vieth, Peter Malfertheiner

**Affiliations:** Department of Gastroenterology, Hepatology and Infectious Diseases, Otto-von-Guericke University, Leipziger Str. 44, Magdeburg, 39120 Germany; Department of Molecular Genetics, Medical Laboratory for Clinical Chemistry, Microbiology and Infectious Diseases, Am Neustädter Feld 47, Magdeburg, 39124 Germany; Institute for Pathology, Otto-von-Guericke University, Leipziger Str. 44, Magdeburg, 39120 Germany; Department for General, Visceral and Vascular Surgery, Otto-von-Guericke University, Leipziger Str. 44, Magdeburg, 39120 Germany; Translational Gastroenterology Unit, Experimental Medicine Division, Nuffield Department of Medicine, University of Oxford, Oxford, UK

**Keywords:** Gastric cancer, mTOR, MMP2, MMP7, MMP9

## Abstract

**Background:**

Regulation of MMP expression by activation of mTOR signalling has been demonstrated for several tumor types, but has thus far not been confirmed in gastric cancer.

**Findings:**

The study compromised 128 patients who underwent gastric resection for cancer (66.4 % male; 86 intestinal, 42 diffuse type). Immunohistochemical staining of MMPs was performed to analyse the topographical pattern of MMP expression at the tumor center and the invasive front, respectively. MMP2 showed higher expression at the invasive front compared to the tumor center, whereas MMP7 staining scores were higher in the tumor center, and there was no difference for MMP9. The expression of p-mTOR was higher in the tumor center than at the invasive front, with a similar trend for mTOR. For intestinal type gastric cancer there was a weak correlation of MMP9 with expression of mTOR in the tumor center. Otherwise, there was no correlation of the MMPs with mTOR. By treatment of MKN45 gastric cancer cells with rapamycin, a reduction of p-mTOR in the Western blot was achieved; however, expression of MMPs remained unaffected.

**Conclusions:**

Expression of MMP2 and MMP7 in gastric cancer is not associated with mTOR, MMP9 expression might be related to mTOR signalling in a subset of tumors.

**Electronic supplementary material:**

The online version of this article (doi:10.1186/s13000-015-0449-z) contains supplementary material, which is available to authorized users.

## Findings

The degradation of the extracellular matrix by matrix metalloproteinases (MMPs) is essential for invasive behaviour of gastric cancer [1–3]. Expression of MMPs can be induced by specific growth factors [4, 5], and depending on the cellular energy level growth factor dependent processes can be regulated by mammalian target of rapamycin (mTOR) related signalling [6]. Thus, also mTOR activation has a role in tumor invasion and metastatic spread [7, 8] and, unsurprisingly, its expression is identified in up to 64 % of gastric cancers [7–9]. The link between growth factor dependent activation of PI3K/Akt/mTOR signalling and downstream up-regulation of MMP gene expression has been shown *in vitro* for several cancer types [4, 10–12]. However, a direct association between mTOR activation and MMP expression has not been shown for gastric cancer so far. MMP2, MMP7 and MMP9 have been most extensively investigated in gastric cancer, but never previously in a direct comparison and in association with mTOR expression. The aim of this study was to investigate whether the expression of MMP2, MMP7, and MMP9 in humans is associated with the expression of mTOR in its “naïve” and its phosphorylated (active) form in different topographical regions of gastric adenocarcinomas. Separate assessment of the tumor center and the invasive front of the cancer has been performed to evaluate the involvement of this potential regulatory mechanism for invasive growth of gastric adenocarcinomas.

**Fig. 1:**
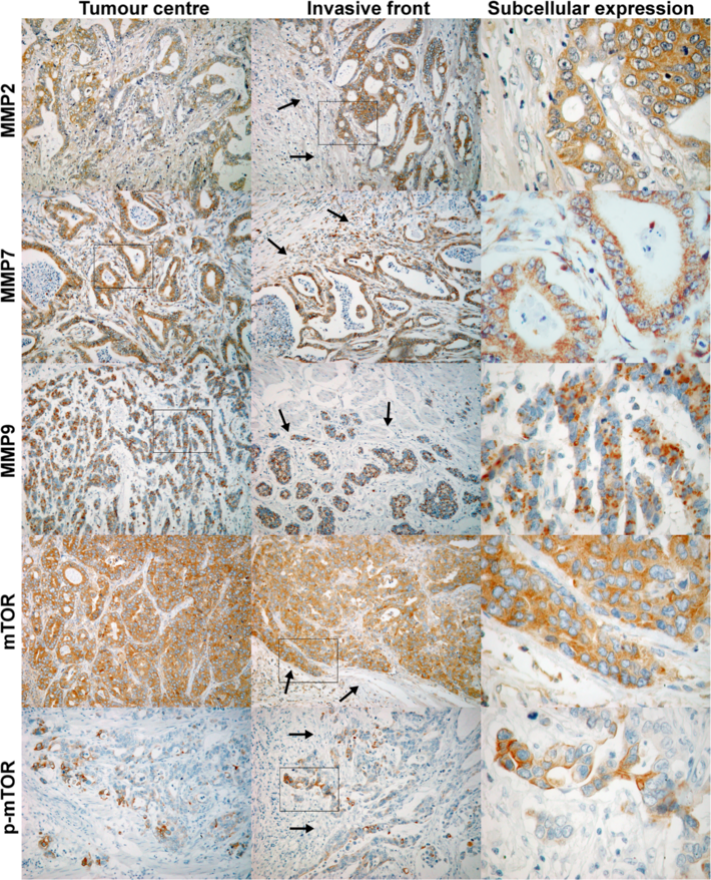
Immunohistochemical staining at the tumor center and the invasive front. Exemplary staining of MMP2, MMP7, MMP9, as well as mTOR and p-mTOR in the tumor center, at the invasive front (100×), and subcellular expression with larger magnification (400×). Arrows are marking the marginal zone of the tumor at the invasive front. Squares are marking the magnified area for each panel

The clinicopathological characteristics of patients who underwent gastrectomy for gastric cancer between 1997 and 2009 were retrospectively identified from the archives of the Magdeburg University Hospital (Table 1). Patients with neoadjuvant treatment and with adenocarcinoma associated with Barrett’s metaplasia and/or location proximal at the esophagogastric junction (Siewert type 1) were excluded from the analysis. For statistical reasons, tumors showing a mixed type according to the Laurén classification (*n* = 14) or cancers with mucinous phenotype (*n* = 2) were combined with the group of diffuse type carcinomas (*n* = 26). Patients with diffuse type gastric cancer below the age of 50 years at diagnosis or who presented with a positive family history were assessed for mutations of the *CDH1* gene, which was negative in all respective cases. Finally, paraffin embedded tissue for immunohistochemistry (IHC) was retrieved for 128 patients (Table 1). The study was approved by the ethics committee of our institution (Ref. 2004–98) and conducted according to the ethical guidelines of the declaration of Helsinki as revised in 1989. In an additional proof-of-principle approach, we measured the expression of *MMP2*, *MMP7* and *MMP9* in MKN45 gastric cancer cells before and after treatment with the mTOR inhibitor rapamycin to investigate the putative link between mTOR signalling and MMP expression in gastric cancer. MMP expression has been assessed by RT-PCR. The activity of mTOR signalling has been assessed by Western blot for the main downstream target of mTOR the P70S6K which is only active in its phosphorylated form (p-P70S6K). Please see Additional file 1 (supplementary methods) for further details. For statistical comparisons, non-parametrical tests have been applied using SPSS 18.0 (SPSS Inc., Chicago, IL, USA). For group comparisons the Mann Whitney U-test was used, Wilcoxon's sign rank test for matched pair comparison between tumor center and invasion front. For correlation analysis Spearman’s rank correlation test was applied. For comparison of categorical data Fisher's exact test was applied. For all tests a two-sided significance level of *p* < 0.05 was considered significant.

**Table 1:** Demographic and clinicopathological characteristics of the main study population

Parameters		Intestinal (*n* = 86)	Diffuse (*n* = 42)	Total (*N* = 128)	p-value
Age in years (median, IQR)		79 (72–85)	78 (68–84)	78 (70–78)	0.239
Sex (male)		61 (70.9 %)	24 (57.1 %)	85 (66.4 %)	0.163
T-stage	T1	13 (15.1 %)	3 (7.1 %)	16 (12.5 %)	0.328
	T2	36 (41.9 %)	15 (35.7 %)	51 (39.8 %)	
	T3	31 (36.0 %)	22 (52.4 %)	53 (41.4 %)	
	T4	6 (7.0 %)	2 (4.8 %)	8 (6.2 %)	
N-stage*	Positive (N1-3)	57 (66.3 %)	39 (92.9 %)	96 (75.0 %)	0.001
M1	Positive (M1)	29 (33.7 %)	20 (47.6 %)	49 (38.3 %)	0.175
Grading*	G1	6 (7.0 %)	0 (0)	6 (4.7 %)	<0.001
	G2	45 (52.3 %)	1 (2.4 %)	46 (35.9 %)	
	G3	35 (40.7 %)	41 (97.6 %)	76 (59.4 %)	
Localisation*	Cardia	30 (34.9 %)	6 (14.3 %)	36 (28.1 %)	0.042
	Corpus	33 (38.4 %)	23 (54.8 %)	56 (43.8 %)	
	Antrum	23 (26.7 %)	13 (31.0 %)	36 (28.1 %)	

Comparison between intestinal and diffuse type tumors was done by Fisher's exact test with significant differences (*p* < 0.05) marked by an asterisk. IQR: interquartile range; Grading: G1: well differentiated, G2: moderately differentiated, G3: poorly differentiated

IHC staining reaction of the tumor was present in 44-60 % for MMP2, MMP7, and MMP9, as well as in 96 % and 80 % for mTOR and p-mTOR, respectively (Additional file 2: Figure S1). As assessed by the immune-reactivity scores [13], only MMP2 was more markedly expressed at the invasive front, whereas MMP9 was homogenously expressed throughout the invasive front and the tumor center (Fig. 1, Table 2). In contrast, MMP7 staining was more pronounced in the tumor center relative to the invasive front. Both mTOR and p-mTOR staining was more pronounced in the tumor center than at the invasive front (Table 2).

**Table 2:** Immune-reactivity score for expression of mTOR, p-mTOR, MMP2, MMP7, MMP9 in the tumor center and at the invasive front of type gastric cancer

Targets	Intestinal type (*n* = 86)			Diffuse type (*n* = 42)			Overall (*N* = 128)		
	Tumor center	Invasive front	p-value	Tumor center	Invasive front	p-value	Tumor center	Invasive front	p-value
MMP2	4.10 ± 5.042	7.65 ± 7.782	<0.001*	2.93 ± 3.925	5.40 ± 6.688	0.005*	3.72 ± 4.721	6.91 ± 7.490	<0.001*
MMP7	5.43 ± 8.345	4.48 ± 7.680	0.008*	5.10 ± 8.798	4.50 ± 7.613	(0.128)	5.32 ± 8.463	4.48 ± 7.628	0.002*
MMP9	4.57 ± 7.362	3.97 ± 7.058	(0.227)	5.62 ± 7.821	4.71 ± 6.656	(0.144)	4.91 ± 7.501	4.21 ± 6.912	(0.097)
mTOR	10.95 ± 7.543	9.79 ± 8.778	0.025*	8.67 ± 6.091	9.12 ± 6.463	(0.589)	10.20 ± 7.156	9.57 ± 8.072	(0.127)
p-mTOR	3.42 ± 4.185	2.69 ± 4.610	0.008*	2.13 ± 2.662	2.76 ± 4.667	(0.545)	3.00 ± 3.792	2.71 ± 4.610	0.013*

Comparison between tumor center and invasive front has been done by the Mann–Whitney U-test with significant differences (*p* < 0.05) marked by an asterisk. Values are given as mean and standard deviation

The immune-reactivity scores for the expression of mTOR and p-mTOR as well as for MMP2, MMP7, and MMP9 in the tumor center correlated each with its expression at the invasive front (*p* < 0.001; Additional file 3: Figure S2a-e). Only for intestinal type tumors, there was a correlation of mTOR with MMP9 expression both in the tumor center (*r* = 0.251, *p* = 0.020) and at the invasive front (*r* = 0.254, *p* = 0.018; Additional file 4: Figure S3a). Otherwise, there was no association between mTOR and MMP2 or MMP7 as assessed by the IHC analysis. Staining of mTOR in the tumor center correlated with p-mTOR (*r* = 0.195, *p* = 0.028; Additional file 4: Figure S3b), and there was a positive association MMP2 with MMP9 at the invasive front (*r* = 0.214, *p* = 0.015; Additional file 4: Figure S3c).

mTOR (*p* = 0.003) and p-mTOR (*p* = 0.02) staining was associated with stage of disease, with lower staining scores in the tumor center of advanced stage cancers (T3 and T4) compared to early disease (T1 and T2). However, the immune-reactivity score for mTOR was higher in the tumor center of patients with evidence of distant metastases (*p* = 0.01), and MMP7 was more highly expressed in the tumors of patients with nodal involvement (tumor center: *p* = 0.01, invasive front: *p* = 0.019; data not shown). Otherwise there was no association of MMP staining with any tumor-associated parameter.

In a parallel proof-of-principle approach, we treated MKN45 gastric cancer cells with rapamycin to investigate the effect of mTOR inhibition on MMP expression. Rapamycin treatment led to an effective inhibition of mTOR signalling, mirrored by a reduction of p-P70S6K, the main downstream target of mTOR signalling (Additional file 5: Figure S4). Transcript levels of *MMP2*, *MMP7* and *MMP9* were clearly expressed in the cells at baseline and were not systematically affected by mTOR inhibition, as assessed by RT-PCR (data not shown).

To our knowledge, this is the first study that analyses the association of the expression of three specific MMPs and mTOR in its native and in its activated, phosphorylated form in human gastric cancer tissue. The expression pattern for MMP2 was consistent with previous reports [14, 15]. Surprisingly, MMP7 showed higher staining scores in the tumor center, but it must be taken into account that we scored only positive staining within the gastric cancer cells and not of stromal components which can also express *MMP7* [16]. Expression of MMP2, MMP7 and MMP9 could be confirmed in the majority of gastric cancers, but there was no significant correlation with the presence of either mTOR or p-mTOR. The association of MMP9 with mTOR was only weak in intestinal type cancers, suggesting a probable interaction of other regulatory mechanisms, such as pathways that respond to inflammatory stimuli [11, 12, 17, 18]. There are further alternative mechanisms that may interfere at this level such as MMP2 being capable of activating MMP9 [19, 20]. In a previous study, *MMP2* and *MMP7* expression were reduced by the mTOR inhibitor rapamycin in human gastric NUGC4 cells that have been stimulated with CXCL12 [21]. MKN45 cells were chosen for this work because that are derived from a poorly differentiated gastric cancer and express both the respective MMPs and mTOR at baseline without the need for stimulation or transfection. Since both mTOR and the MMPs were expressed in MKN45 and the negative findings supported the IHC data showing no association between mTOR and MMP expression in gastric cancer no additional cell line validation was undertaken. The lack of an association of mTOR signalling and MMP expression might be due to the interplay with pathways that are involved in regulation of mucosal inflammation which are dependent on the very complex tissue microenvironment within gastric cancer. As mentioned above for MMP9, NFκB-dependent induction in response to inflammatory stimuli has been reported [11, 17, 18, 20, 22, 23], and *MMP9* expression is raised in cag-dependent manner in the course of *H. pylori* infection. *H. pylori* induced inflammation can induce both *MMP9* and *MMP7* expression in the gastric mucosa mediated by tissue macrophages [24–27]. Furthermore, mTOR seems to have a stage-dependent impact, with the expression being higher in earlier stages of gastric carcinogenesis; however, our cohort mostly consisted of advanced stage cancers.

It has been reported that expression and functional activity of both mTOR and specific MMPs are associated with less favourable prognosis in gastric carcinoma, with overall heterogeneous results and stronger evidence for mTOR [3, 7, 28–31]. None of the parameters analysed were associated with overall survival in our cohort (data not shown). However, due to the retrospective nature of the study, survival data could not be gathered for all patients.

In summary, an association between the presence of MMP2 and MMP7 proteins and (p-)mTOR expression in gastric cancer could not be confirmed. A correlation of MMP9 with mTOR expression in intestinal type cancers was only of weak character and doesn't support mTOR as being the main regulating factor. Future studies should address alternative pathways and consider the influence of the inflammatory microenvironment of the surrounding mucosa.

## Additional files

Additional file 1: (DOCX 25 kb) Additional file 2: Figure S1. Relative expression of mTOR and MMPs. Partition of samples with positive staining of mTOR, p-mTOR, MMP2, MMP7 and MMP9 each at the tumor center and at the invasive front. Comparison of both localisations have been done by Fisher's exact test. Significant differences (p<0.05) are marked with an asterisk. (TIF 447 kb) Additional file 3: Figure S2. Correlation of staining scores between tumor center and invasive front. Displayed are the patient-matched paired IRS for the staining reaction at the tumor center and the invasive front for (a) MMP2, (b) MMP7, (c) MMP9, (d) mTOR and (e) p-mTOR. Correlation of the IRS for tumor center and invasive front was done by Spearman's rank correlation test with p<0.05 considered as significant. (TIF 4658 kb) Additional file 4: Figure S3. Correlation of staining scores between different factors. Displayed are the patient-matched paired IRS for MMP9 and mTOR at the tumor center of intestinal type cancers (a), for mTOR and p-mTOR at the tumor center (b), and for MMP2 and MMP9 at the invasive front (c). Correlation analysis was done by Spearman's rank correlation test with *p*<0.05 considered as significant. (TIF 1937 kb) Additional file 5: Figure S4. mTOR inhibition by rapamycin in MKN45 gastric cancer cells. Western Blot of expression of p-P70S6K1, P70S6K1 and β-actin in MKN45 cells that have been treated with rapamycin (RAPA) for each 24h, 48h and 72h (a). Corresponding results of the PCR analysis of the transcript content MMP2, MMP7 and MMP9. There was no significant effect on MMP expression by treatment with rapamycin. For MMP9 only assessment after 48 hours gave consistent results. UC: untreated controls; DMSO: treatment with DMSO only. (TIF 2341 kb)

## Abbreviations

*H. pylori*: *Helicobacter pylori*
IHC: immunohistochemistry
IRS: immune-reactivity score
MMP: matrix metalloproteinase
mTOR: mammalian target of rapamycin
RT-PCR: reverse transcription (real time) polymerase chain reaction
