# Supplementary material for: MMP2 and MMP7 at the invasive front of gastric cancer are not associated with mTOR expression
Source: Diagn Pathol. 2015 Dec 12;10:212. doi: 10.1186/s13000-015-0449-z (PMC4676863; doi:10.1186/s13000-015-0449-z)
Supplement: Additional file 1: — (DOCX 25 kb) [file 13000_2015_449_MOESM1_ESM.docx]

**Supplementary Methods**

***Immunohistochemistry***

3µm sections from formalin-fixed and paraffin-embedded tissue were incubated overnight at 60°C followed by stepwise rinsing in Xylol and ethanol dilution series. Slides were heated for 10 seconds at 120°C in 0.01M EDTA buffer using a steam-pressure chamber. Prior to staining for MMPs, slides were treated with Proteinase 1 (Ventana Medical Systems, Inc., Munich, Germany), and prior to mTOR and p-mTOR staining pre-treatment with citrate was used. The primary antibodies used included: MMP2 Mouse IgG1k (1:100 dilution), MMP7 Mouse IgG2b (1:50 dilution), MMP9 Mouse IgGk (1:100 dilution), all from Millipore BioSciences, Temecula, CA, USA. Additional antibodies used were: mTOR (7C10) Rabbit IgG (dilution 1:30) and phospho-mTOR(Ser2448) (49F9) Rabbit IgG (1:50 dilution) from Cell Signaling Technology, Inc., Danvers, MA, USA.

The primary antibodies were applied at 37°C for 32 minutes. Automated staining used the NexES IHC staining module (Ventanta Medical Systems, Inc., Munich, Germany), the iVIEW DAB Detection Kit (Ventana Medical Systems, Inc., Munich, Germany) and the indirect biotin-streptavidin method before counterstaining with Haemalaun solution. Specificity of the staining was tested by selective substitution of the primary antibody by non-immunogenic serum.

Semiquantitative evaluation of the staining reaction was undertaken using the modified immune-reactivity score (IRS) (16). The partition of positively stained cells (PP: 0-100%, represented by scores 0-10 with 0=0%,1=10% and 10=100%) was multiplied with the intensity of staining (SI: 0-3; 0=negative, 1=weak, 2=moderate, 3=strong reaction), resulting in a score ranging from 0 to 30. Only staining of tumor cells has been scored for this study and scoring has been applied separately for each patient for the tumor center respresented by the main bulk of malignant cell conglomeration, and for the invasive front, respresented by scattered malignant cells at the border of the adjacent tissue structures, mostly stromal compartments.

***Cell culture of MKN45 cells***

MKN45 cells are generated from a poorly differentiated gastric adenocarcinoma (73). Cell culture was performed in 75cm^3^ cell culture flasks (Nunclon^TM^, Nunc, Roskilde, Denmark) in RPMI medium 1640 with L-Glutamin, and addition of 10% fetal calf serum, 1% penicillin, and 1% gentamycin (all PAA Laboratories GmbH, Cölbe, Germany) at 37°C and 5% CO_2_. Medium was changed three times per week. After rinsing in 10ml PBS (Dulbecco’s 1x Phosphate buffered saline, PAA Laboratories GmbH, Cölbe, Germany), trypsin was applied for solution of confluent colonies in 1:10 PBS for 5 minutes in 37°C, and the cells suspended in 10ml medium before transfer in Greiner tubes (Greiner Bio-One GmbH, Frickenhausen, Germany). The suspension was centrifuged for (10minutes, 800x g, Hereaus Instruments, Hanau, Frankfurt, Germany) and the supernatant discarded, before re-suspension of the cell pellet in 20ml medium. Cell count was performed on an aliquot that was stained with trypan blue (Roth, Karlsruhe, Germany) under the microscope (10x magnification) using a Neubauer counting chamber (Brand, Wertheim, Germany). 500000 cells were plated on a 6 well plate (Nunclon^TM^, Nunc, Roskilde, Denmark) and medium then added up to a total volume of 2ml. Cells were incubated for 24h at 37°C before further processing.

***MKN45 cell processing***

Cells were treated with 100nM rapamycin and incubated for each 24h, 48h, or 72h at 37°C in 5% CO_2_. Each assay was run in three replicates. For each run two controls were included, one each untreated and one with DMSO only. For RNA extraction, the supernatant was discarded and the adherent cells washed in 1x PBS before 350µl RLT buffer with 10% Mercaptoethanol (ApploChem GmbH, Darmstadt, Germany) was applied. Cells were mobilised using a cell scratcher (TPP Techno Plastic Products AG, Trasadingen Switzerland), transferred into an Eppendorf tube. The RNeasy Mini Kit^TM^ (Qiagen, Hilden, Germany) was used for RNA extraction including DNAse treatment to prevent DNA pollution. Quantification, cDNA synthesis and qRT-PCR were performed according to the methods mentioned for tissue samples.

For protein extraction, the supernatant was discarded and the adherent cells washed in 1x PBS before 150µl protein lysis buffer including proteinase inhibitor was added. Cells were mobilised using a cell scratcher (TPP Techno Plastic Products AG, Trasadingen, Switzerland), transferred into an Eppendorf tube and put on ice for 1h before cell destruction via ultrasound application for 10s (UP 200H, Dr.Hielscher GmbH, Teltow, Germany). The lysate was centrifuged for 10min at 14000x g, 4°C, and 120µl of the supernatant stored at -20°C. Protein concentration analysis was performed using the RC DC^TM^ Protein Assay Reagents Package (Bio-Rad Laboratories GmbH, Munich, Germany) according to the manufacturer’s instructions. For photometric assessment the Microplate Reader Synergy HT (BioTek, Bad Friedrichshall, Germany) was used at a wavelength of 750nm. Protein content of mTOR and p-mTOR in cell lysates were assessed by Western blotting (supplementary methods), qRT-PCR was used for quantification of MMP2, MMP7 and MMP9.

***RNA extraction and quantitative RT-PCR***

RNA extraction was performed using the Qiagen RNeasy Plus Universal Mini Kit 73404 (Qiagen, Hilden, Germany) according to the single step method (45). RNA content was quantified UV-spectrometric at a wavelength of 260nm using the Pharmacia Biotech GeneQuant^TM^ (GE Healthcare, Munich, Germany). The purity of RNA was assessed by the ratio of the optical density (OD) at 260nm versus 280nm wave length (OD_260_/OD_280_: 1.8-2.2). cDNA synthesis was performed for 45 minutes at 42°C on a Thermomixer (Eppendorf, Hamburg, Germany) using 2000ng RNA in 25.4µl RNAse-free water and 14.6µml mastermix (1.0µl Recombinant-Rnasin Ribonuclease Inhibitor, 8.0µml AMV-RT 5x reaction buffer, 2.0µl AMV reverse transcriptase, each Promega, Mannheim, Germany; 1.6µl dNTP-mix (10mM), Peqlab Biotechnologie GmbH, Erlangen, Germany; 2.0µl Random-Primer (0.04 A260U/µl), Roche Diagnostics, Mannheim, Germany). Inactivation of the reverse transcriptase was achieved by heating to 95°C for 5 minutes.

Quantitative RT-PCR was performed using a BIO-RAD C1000^TM^Thermalcycler (BioRad Laboratories GmbH, Munich, Germany). The reaction mixture consisted of 15 µL 2x Quanti-Tect RSYBR-Green Mastermix R (Qiagen, Hilden, Germany), 13.4µl RNase-free water, 0.2µl of both forward (fw) and reverse (rev) primer for each gene, and 1.2 µL cDNA (40 cycles, annealing temperature 60°C for 30sec, for *MMP7* annealing temperature 60.6°C for 30sec, incubation 95°C, elongation 72° for 30sec).

The following primers were used for the qRT-PCR analysis: *MMP2* (fw: 5’-gcctttgctcgtgccttccaag-3’; rev: 5’-cggcgttcccatacttcacacg-3’), *MMP7* (fw: 5’-gatgtggagtgccagatgttgc-3’; rev: 5’-agcatctcctccgagacctgtc-3’), *MMP9* (fw: 5’-ccaaacctttgagggcgacctc-3’; rev: 5’-tcatcgtcgaaatgggcgtctc-3’), *β-actin* (fw: 5’-catgccatcctgcgtctggacc-3’, rev: 5’-acatggtggtgccgccagaca-3’). The results were normalized to *β-actin*.

***Western Blot***

According to the overall protein concentration, samples were diluted with 4x Lämmli buffer to generate a final protein concentration of 15µg per well before incubated for 5min at 95°C in loading buffer and then being transferred in the collection gel. Protein separation was achieved by application of 85V at the collection gel and 120V at the separation gel for 2.5h in a electrophoresis chamber (Biostep, Jahnsdorf, Germany) filled with running buffer (200ml 10x Tris-Glycin-buffer, 20mol 10% SDS, in 2l Aqua dest.). Page ruler Prestained Protein Ladder (Fermentas GmbH, St. Leon-Rot, Germany) was used as marker for the molecular weight. After electrophoresis the separation gel was transferred on the nitrocellulose membrane (PVDF Transfer Membrane, Thermo Fisher Scientific, Rockford, USA) that has been activated for 1min in Methanol. Protein bands were transferred from the gel to the membrane by application of 100V for 1h in a cooled transfer chamber (Biostep, Jahnsdorf, Germany) filled with transfer buffer (200ml 10x Tris-Glycin-buffer, 400ml 20% Methanol, in 2l Aqua dest.). The membrane was blocked with 5% milk-TBS-T for 30 minutes to prevent binding of unspecific antigens. The primary anti-S6K(phosphor T389)-antibody (ab2571, Abcam plc., Cambridge, UK) was suspended in 3% BSA in TBS-T (1:500 dilution), the primary anti-S6K-antibody (ab9366, Abcam plc, Cambridge, UK) was suspended in 5% milk-TBS-T (1:200 suspension), both suspensions were incubated at 4°C overnight on the shaker. The membrane was then washed four times in 1x TBS-T before incubation with the secondary antibody (1:5000 dilution, Goat-Anti-Rabbit-IgG peroxidase-conjugated, Dianova, Hamburg, Germany) in 5% milk-TBS at room temperature. After additional washing in 1% TBS-T 2000µl substrate (SuperSignal® West Dura, Extended Duration Substrate, Thermo Fisher Scientific, Rockford, USA) was added for the peroxidase staining reaction. Development of the staining was done using the INTAS chemi-luminescence device for an increasing time series form 10s to 5min (INTAS, Science Imaging Instruments GmbH, Göttingen, Germany) before the membrane was washed again in 1x TBS-T and the primary and secondary antibodies were removed by Restore^TM^ PLUS Western Blot Stripping Buffer (Thermo Fisher Scientific, Waltham, USA). The membrane was blocked again for 30min with 5% milk-TBS-T and the primary β-actin antibody (1:10000 dilution, Monoclonal Anti-β-actin antibody, Sigma Aldrich Chemie GmbH, Steinheim, Germany) in 3% BSA-TBS-T was applied at 4°C overnight. Finally, the secondary antibody (1:80000 dilution, Anti-mouse-IgG-peroxidase-AK, Sigma Aldrich Chemie GmbH, Steinheim, Germany) in 5% milk-TBS-T was applied for 1h at room temperature before development of the membrane.
