# Supplementary figures and images for: MMP2 and MMP7 at the invasive front of gastric cancer are not associated with mTOR expression
Source: Diagn Pathol. 2015 Dec 12;10:212. doi: 10.1186/s13000-015-0449-z (PMC4676863; doi:10.1186/s13000-015-0449-z)

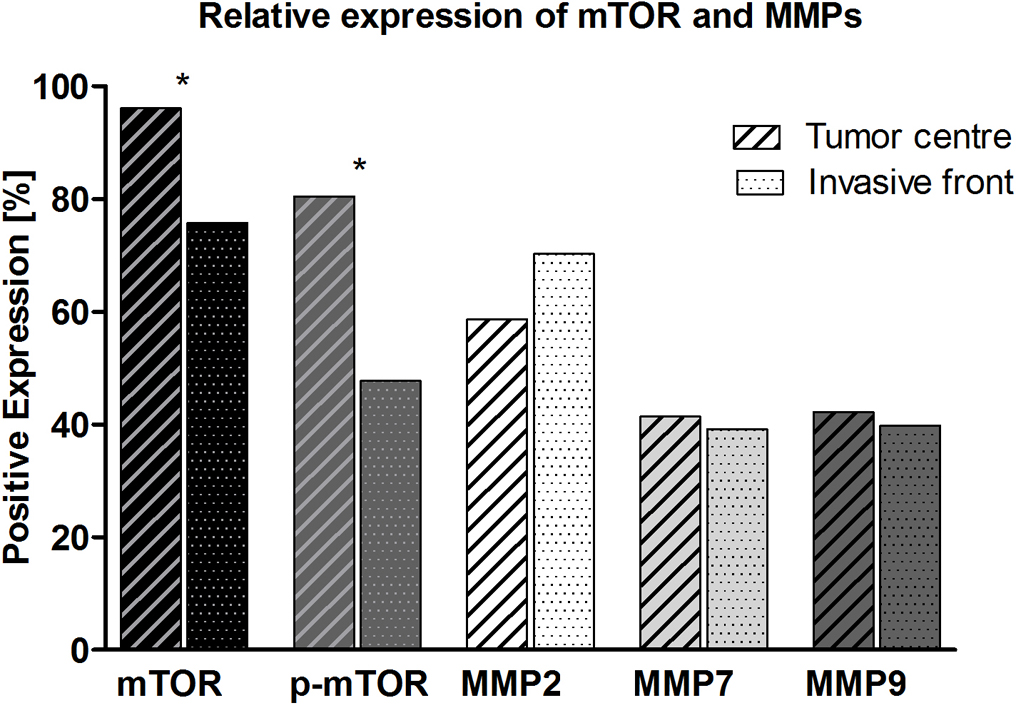

Supplement: Additional file 2: Figure S1. — Relative expression of mTOR and MMPs. Partition of samples with positive staining of mTOR, p-mTOR, MMP2, MMP7 and MMP9 each at the tumor center and at the invasive front. Comparison of both localisations have been done by Fisher's exact test. Significant differences (p<0.05) are marked with an asterisk. (TIF 447 kb) [file 13000_2015_449_MOESM2_ESM.tif]

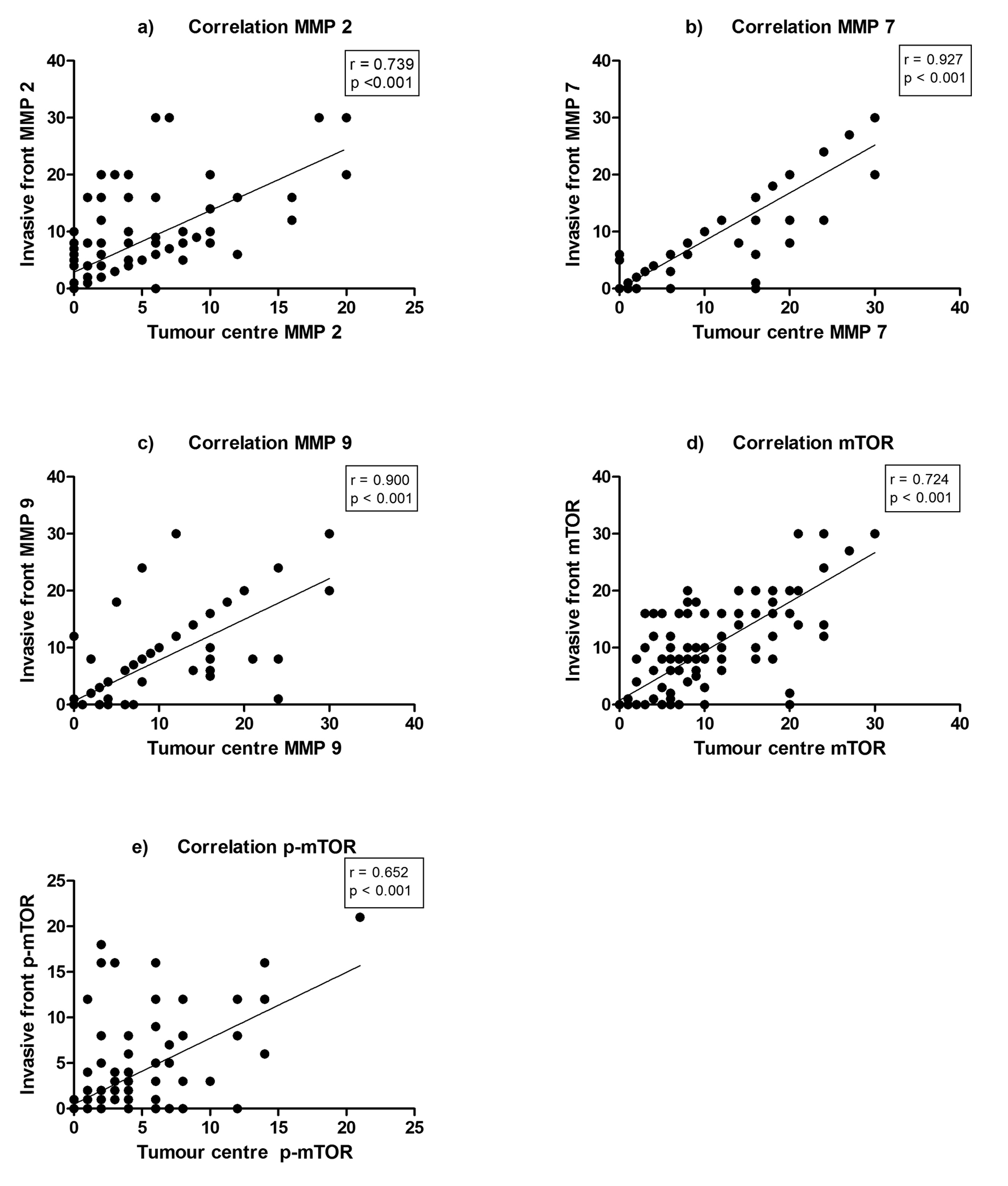

Supplement: Additional file 3: Figure S2. — Correlation of staining scores between tumor center and invasive front. Displayed are the patient-matched paired IRS for the staining reaction at the tumor center and the invasive front for (a) MMP2, (b) MMP7, (c) MMP9, (d) mTOR and (e) p-mTOR. Correlation of the IRS for tumor center and invasive front was done by Spearman's rank correlation test with p<0.05 considered as significant. (TIF 4658 kb) [file 13000_2015_449_MOESM3_ESM.tif]

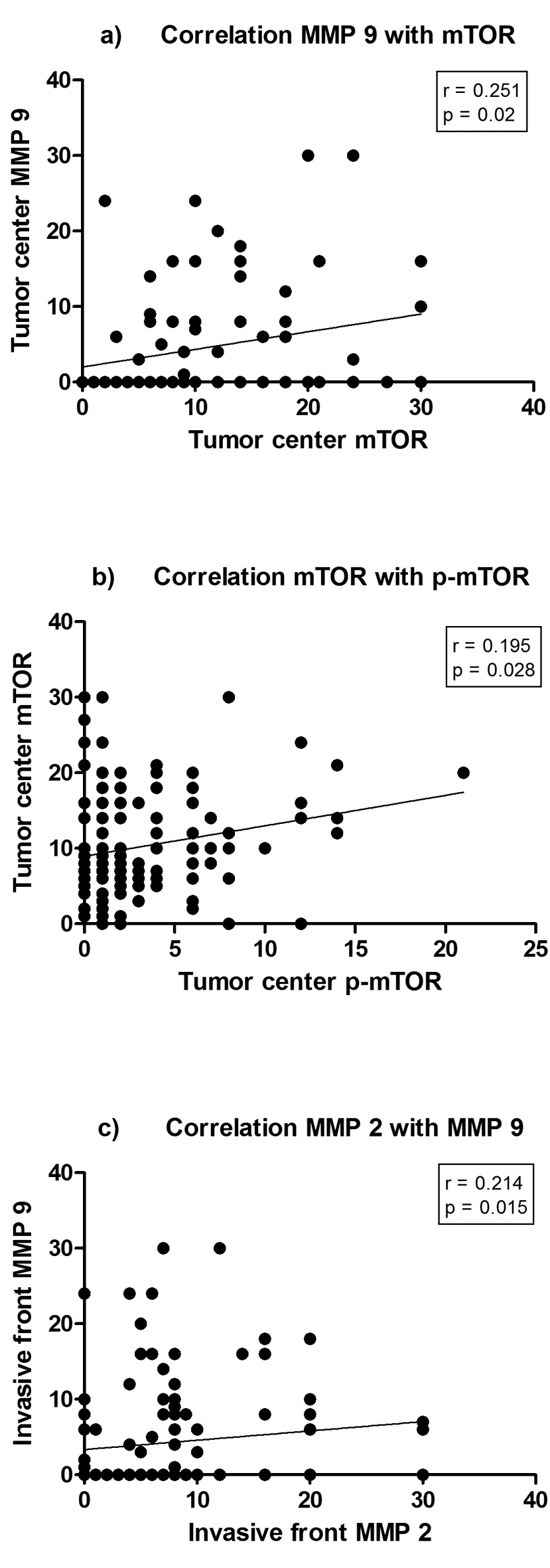

Supplement: Additional file 4: Figure S3. — Correlation of staining scores between different factors. Displayed are the patient-matched paired IRS for MMP9 and mTOR at the tumor center of intestinal type cancers (a), for mTOR and p-mTOR at the tumor center (b), and for MMP2 and MMP9 at the invasive front (c). Correlation analysis was done by Spearman's rank correlation test with p<0.05 considered as significant. (TIF 1937 kb) [file 13000_2015_449_MOESM4_ESM.tif]

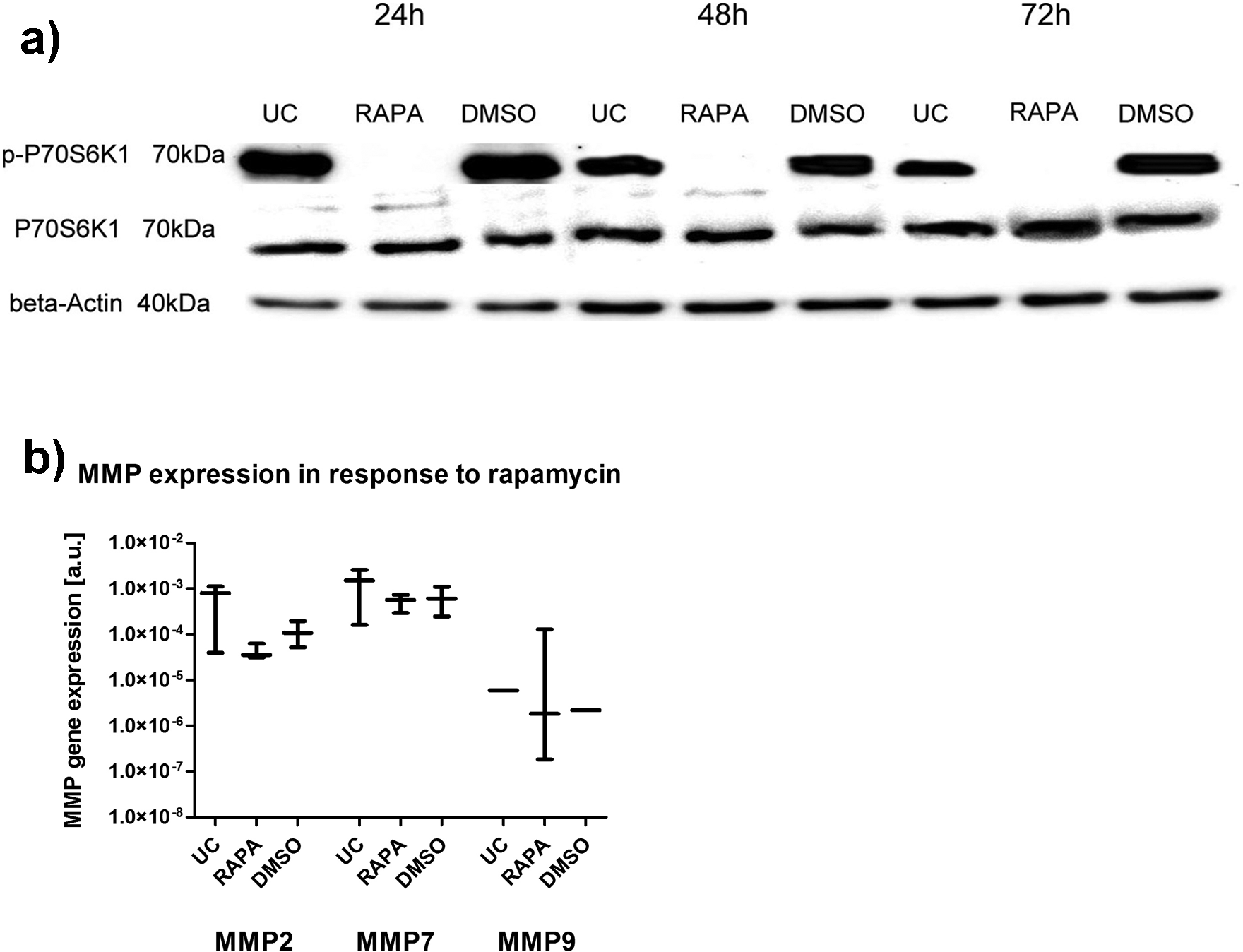

Supplement: Additional file 5: Figure S4. — mTOR inhibition by rapamycin in MKN45 gastric cancer cells. Western Blot of expression of p-P70S6K1, P70S6K1 and β-actin in MKN45 cells that have been treated with rapamycin (RAPA) for each 24h, 48h and 72h (a). Corresponding results of the PCR analysis of the transcript content MMP2, MMP7 and MMP9. There was no significant effect on MMP expression by treatment with rapamycin. For MMP9 only assessment after 48 hours gave consistent results. UC: untreated controls; DMSO: treatment with DMSO only. (TIF 2341 kb) [file 13000_2015_449_MOESM5_ESM.tif]
